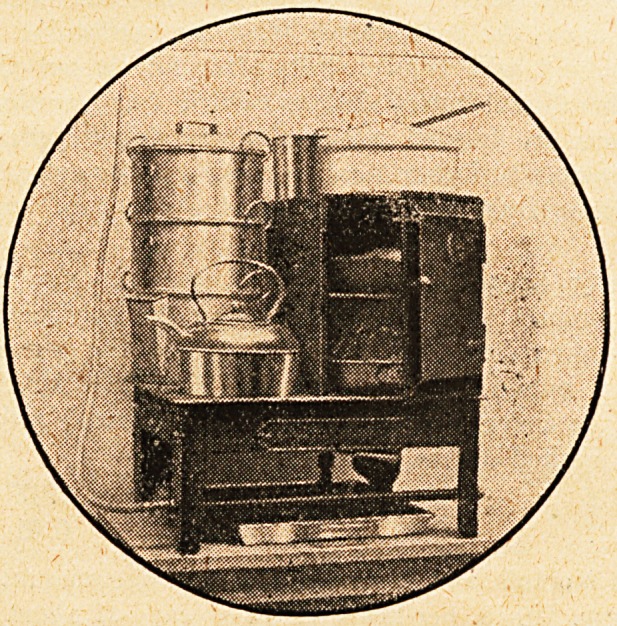# Labour-Saving Appliances

**Published:** 1917-04-21

**Authors:** 


### LABOUR-SAVING APPLIANCES.

### MODERN GRATES.

Who has not frowned over the old-fashioned fireplace, burning up a tremendous amount of fuel, whilst a large proportion of the heat produced disappears up the chimney? This is not the only disadvantage of the old grates; they are also difficult to keep tidy, and are very dusty. An entirely new grate is expensive, but there are now on the market some excellent contrivances which

The "Bewty" Fire Front.

Grate fitted with "Bewty" Fire.

The Old Method.

The "Bewty" Fire.

can be fitted on to the existing fireplaces at little cost, and this would soon be refunded by the saving of coal.

These contrivances are the "Cosy," costing from 9s 6d. upwards, made by the Falkirk Iron Company, Craven House, Kingsway, W.C., and the "Bewty," made by the Interoven Stove Company, 156 Charing Cross Road, W.C., the prices of the latter being from 22s. 6d. each. Both kinds can be fitted with the greatest ease, and can be used in any grate from which the bars can be removed, converting it into a modern and very presentable stove, and at the same time fulfilling the necessity for economy.

### SCRUBBING WITHOUT KNEELING.

A very useful appliance for use for scrubbing is sold by the Staines Kitchen Equipment Company, Ltd., 141 Victoria Street, S.W. This consists of a pail with a perforated stand fixed to it, in which a mop is squeezed. This mop takes the place of the ordinary house-flannel, and instead of the ordinary scrubbing-brush a hard brush is fixed on a long handle, so that scrubbing of rooms, passages, and stairs can be done without putting the hands into water, and in an upright position. It is claimed that this is a much quicker method than the old one. A matron of a Mothers' Welcome said that the rooms, staircase, and passages used by the mothers could all be scrubbed down in twenty minutes with this contrivance, whereas with the scrubbing-brush and house-flannel the same work took half a day, and the result did not equal the quicker way.

### A HANDY COOKER.

A very useful gas-saving contrivance, suitable for a ward kitchen or, indeed, for large kitchens, is the "One Ring" table cooker. A stand or hot-plate with legs, the whole made of cast iron, can be put over a gas ring, and saucepans, kettles, etc., put on the hot-plate, or this same stand can be used with a "Primus" stove. This cooker is supplied by the London Warming and Ventilating Company, 20 Newman Street, Oxford Street, W.

## Figures and Tables

**Figure f1:**
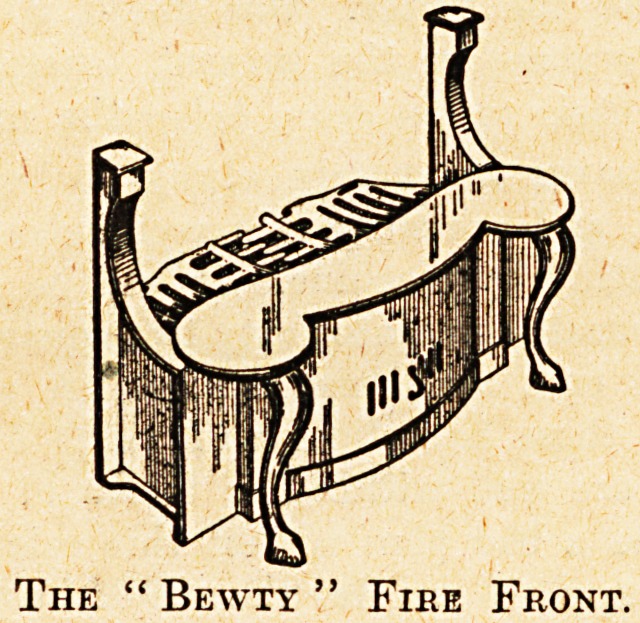


**Figure f2:**
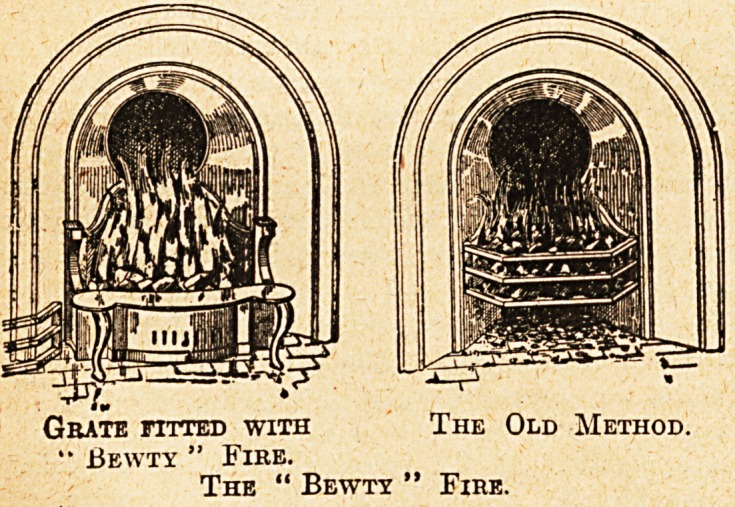


**Figure f3:**